# Regulation of Cancer Aggressive Features in Melanoma Cells by MicroRNAs

**DOI:** 10.1371/journal.pone.0018936

**Published:** 2011-04-25

**Authors:** Eyal Greenberg, Liat Hershkovitz, Orit Itzhaki, Steven Hajdu, Yael Nemlich, Rona Ortenberg, Nir Gefen, Liat Edry, Shira Modai, Yona Keisari, Michal J. Besser, Jacob Schachter, Noam Shomron, Gal Markel

**Affiliations:** 1 Ella Institute of Melanoma, Sheba Medical Center, Tel Hashomer, Israel; 2 Department of Clinical Microbiology and Immunology, Sackler Faculty of Medicine, Tel Aviv University, Tel Aviv, Israel; 3 Cancer Research Center, Sheba Medical Center, Tel Hashomer, Israel; 4 Department of Cell and Developmental Biology, Sackler Faculty of Medicine, Tel Aviv University, Tel Aviv, Israel; 5 Talpiot Medical Leadership Program, Sheba Medical Center, Tel Hashomer, Israel; University of Bergen, Norway

## Abstract

MicroRNAs (miRNAs) are small non-coding RNAs with regulatory roles, which are involved in a broad spectrum of physiological and pathological processes, including cancer. A common strategy for identification of miRNAs involved in cell transformation is to compare malignant cells to normal cells. Here we focus on identification of miRNAs that regulate the aggressive phenotype of melanoma cells. To avoid differences due to genetic background, a comparative high-throughput miRNA profiling was performed on two isogenic human melanoma cell lines that display major differences in their net proliferation, invasion and tube formation activities. This screening revealed two major cohorts of differentially expressed miRNAs. We speculated that miRNAs up-regulated in the more-aggressive cell line contribute oncogenic features, while the down-regulated miRNAs are tumor suppressive. This assumption was further tested experimentally on five candidate tumor suppressive miRNAs (miR-31, -34a, -184, -185 and -204) and on one candidate oncogenic miRNA (miR-17-5p), all of which have never been reported before in cutaneous melanoma. Remarkably, all candidate Suppressive-miRNAs inhibited net proliferation, invasion or tube formation, while miR-17-5p enhanced cell proliferation. miR-34a and miR-185 were further shown to inhibit the growth of melanoma xenografts when implanted in SCID-NOD mice. Finally, all six candidate miRNAs were detected in 15 different metastatic melanoma specimens, attesting for the physiological relevance of our findings. Collectively, these findings may prove instrumental for understanding mechanisms of disease and for development of novel therapeutic and staging technologies for melanoma.

## Introduction

Melanoma, an aggressive malignancy arising from melanocytes, is one of the main life-threatening malignancies of our era. While it accounts for nearly 4% of all skin cancers, it causes 75% of skin cancer–related deaths worldwide and is considered to be the most common fatal malignancy of young adults [1]. Transformation and development of metastasis require stepwise acquisition of aggressive characteristics. These include, for example, uncontrolled growth, resistance to apoptosis, motility, proteolytic capacity and adhesion (reviewed in [2], [3]). In addition, plasticity of melanoma cells is evident by their ability to form tube-like structures [4]. These functional vascular-like structures are comprised of tumor cells [5] and their presence is associated with poor prognosis [6], [7]. Recent development of targeted therapy for melanoma emphasizes the importance of molecular delineation of the underlying mechanisms of pathogenesis [8].

MicroRNAs (miRNAs) are small, non-coding, 19–22 nucleotide long RNA molecules, which function as specific epigenetic regulators of gene expression by inhibiting protein translation, leading mRNA to degradation, or both [9], [10]. Once processed from their distinctive hairpin transcripts and loaded into the Argonaute protein of the silencing complex, the miRNAs pair with cytoplasmic mRNA to direct posttranscriptional repression. The “seed” region, which is found between nucleotides 2 to 8 of the mature miRNA, binds to complementary regions in the 3′ un-translated region (3′-UTR) of target mRNA. To date, close to 1000 human miRNAs have been identified [11], which are thought to regulate at more than 50% of human genes [12].

miRNAs are involved in the regulation of many biological processes, such as embryonic development, cell differentiation, cell cycle, apoptosis and angiogenesis (reviewed in [13]). They are also directly implicated in cancer development, progression and metastasis *in-vitro*, *in-vivo* and reported even in patients [10], [14]. In some cases, cancer is facilitated by the loss of certain miRNAs, such as miR-15/16 cluster in chronic lymphocytic leukemia [15], miR-34a in uveal melanoma [16] and miR-31 in mesothelioma [17]. The loss of these miRNAs enhances invasiveness, migration and proliferation of cancer cells. In other cases, cancer is facilitated by the over-expression of other miRNAs, such as miR-17-92 cluster [13], [18], which promotes migration and invasion in several malignancies.

Currently, our knowledge on the roles of miRNAs in melanoma development and progression is still limited. Recently, several comparative miRNA profiling studies of normal melanocytes and melanoma cells revealed: 1) Groups of miRNAs associated with malignant transformation, progression and metastatic potential [19]; 2) Specific expression profiles that were associated with mutational status and survival [20]; 3) Differential miRNA patterns in melanoma of young adults and older adults [21]; and 4) Prediction of post-recurrence survival [22]. Yet none of these studies described miRNAs that directly determine aggressive features of cutaneous melanoma, such as enhanced proliferation, motility and invasion. Few inhibitory miRNAs were identified in melanoma, including miR-34a (uveal melanoma) [16], miR-193b [23], let-7a [24], and miR-211 [25], [26], while miR-182 [27] and miR-221/222 [28]were shown to stimulate metastatic potential of melanoma cells. Given the critical evaluation of aggressive versus not aggressive melanoma, and the potential of therapeutics, we find it imperative to learn the molecular events of aggressive melanoma.

Here we focus on high-throughput identification of miRNAs that are directly involved in determination of an aggressive melanoma cell phenotype. Two isogenic melanoma cell-lines with a different aggressive pattern, the highly aggressive C8161 cells and the poorly aggressive C81-61 cells, were subjected to differential high-throughput screening of miRNAs. We hypothesized that due to the isogenic background of the cells, the differentially expressed miRNA groups would be enriched for miRNAs with a direct effect on aggressive melanoma features. Indeed, we provide experimental evidence *in-vitro*, *in-vivo* and in clinical melanoma specimens that previously known tumor-suppressive and tumor-promoting miRNAs fit this hypothesis. Remarkably, we describe new miRNAs with little information regarding their role in cancer, such as miR-185. The scientific and translational implications of this study are discussed.

## Results

### Differential aggressiveness of melanoma isogenic cell lines

**Figure 1:**
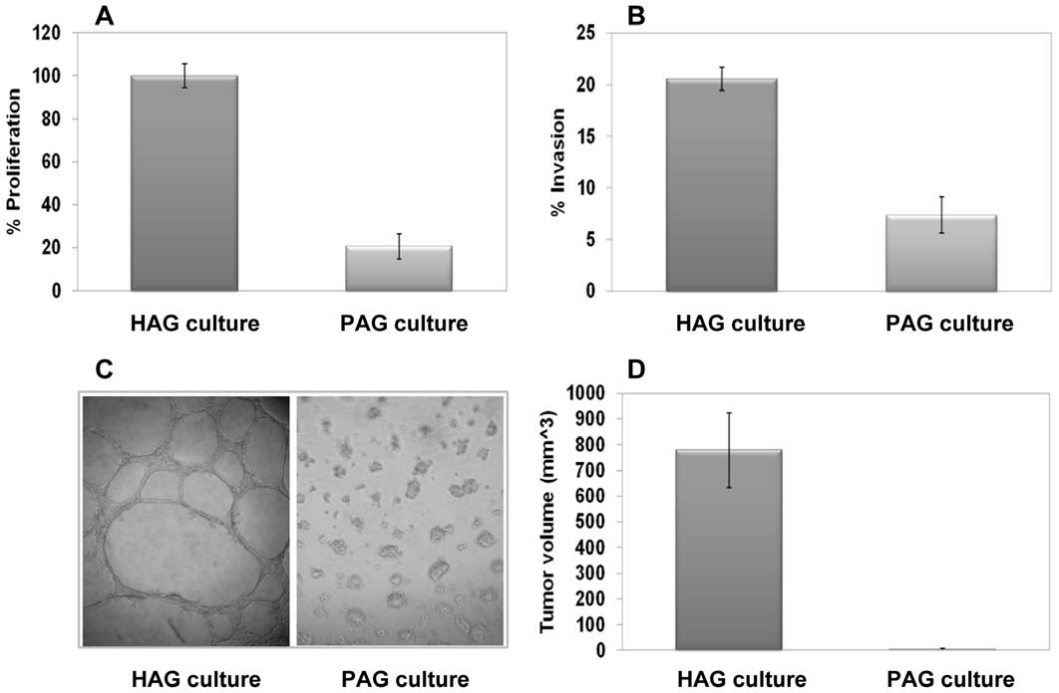
Differential aggressiveness between two isogenic melanoma lines. (A) Proliferation was determined with standardized XTT colorimetric assay 24 h after seeding. The number of HAG cells was determined as 100%. Figure shows a representative experiment out of three performed; (B) The percentage of invading cells tested in a 20 h matrigel invasion assay. Results were corrected for proliferation rate. Figure shows a representative experiment out of three performed; (C) HAG cells or PAG cells were tested for tube formation activity. Representative whole field microphotographs are shown from a representative experiment, out of three performed; (D) The average tumor masses formed in SCID-NOD mice 30 days post injection of 1×10^6^ cells of HAG or PAG cell lines. Each group included at least six mice.

The highly aggressive (HAG) C8161 and poorly aggressive (PAG) C81-61 cutaneous melanoma cell lines were derived from different metastases from the same patient [29]. The differential aggressive phenotype of the HAG and PAG cells was confirmed by four different functional assays: proliferation, invasion through matrigel, tube formation in matrigel and formation of tumors in xenografted mice. Indeed, HAG cells displayed substantially higher proliferation, invasion and tube formation capabilities *in-vitro*, than PAG cells (Figure 1, A–C). Moreover, subcutaneous injection of 1×10^6^ HAG cells formed tumors in SCID-NOD mice within five days post injection, with an average xenograft size of 780 mm^3^ by day 30. In contrast, injection of 1×10^6^ PAG cells did not develop into measurable tumors within 30 days (Figure 1D). Small PAG tumors (∼200 mm^3^) were observed >90 days post injection of PAG cells (data not shown).

### Differential expression profile of miRNAs among HAG and PAG cells

**Figure 2:**
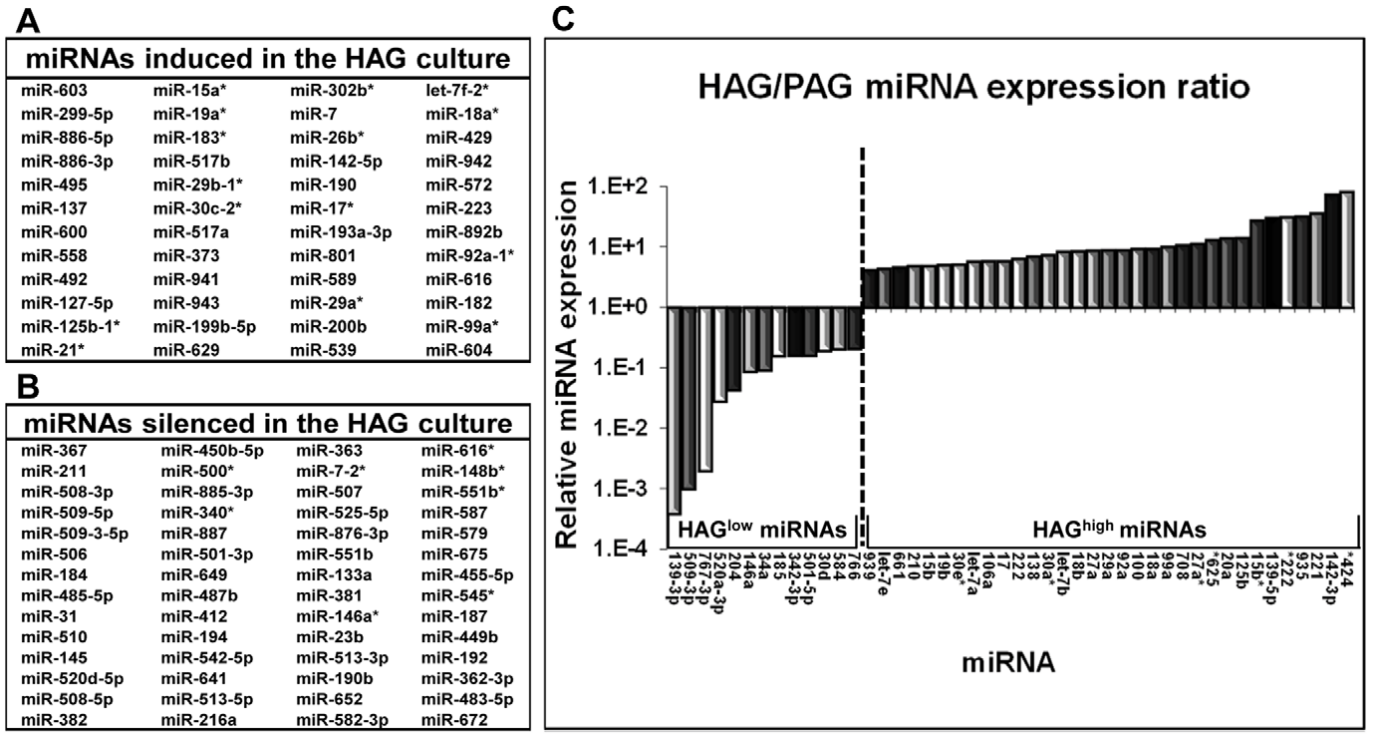
Differential miRNA expression profile in HAG and PAG cells. (A) The list of miRNAs that are expressed in the HAG cells, but not in the PAG cells; (B) The list of miRNAs that are expressed in the PAG cells, but not in the HAG cells; (C) Relative expression of miRNAs that are expressed in both HAG and PAG cells. Expression of HAG-to-PAG ratio (Y-axis) is shown as 2∧-ΔΔCt. HAG^low^ represents miRNAs with low HAG-to-PAG ratio. HAG^high^ represents miRNAs with high HAG-to-PAG ratio.

The remarkable phenotypic difference between the two isogenic melanoma lines comprised the platform for studying epigenetic regulation of aggressive features by miRNAs. A comparative high-throughput profiling of miRNAs was performed. Importantly, a set of 81 miRNAs were expressed at higher levels in the HAG as compared to the PAG cells (HAG^high^). Another set of 69 miRNAs was expressed at lower levels in the HAG as compared to the PAG cells (HAG^low^). A group of 48 miRNAs out of the 81 HAG^high^ miRNAs were not expressed at all in the PAG cells (Figure 2A), while 56 miRNAs out of the 69 HAG^low^ miRNAs were not detected in HAG cells (Figure 2B). The rest of the differentially expressed miRNAs were found in both cell lines at different, quantifiable, amounts (Figure 2C). We hypothesized that these miRNA clusters would be enriched for miRNAs that are involved in direct regulation of the distinct phenotypic differences. More specifically, we speculated that the HAG^low^ miRNAs would be enriched in miRNAs accounting for suppressive effects on various cell features e.g. proliferation (Suppressive miRNAs), while the HAG^high^ miRNAs would be enriched in miRNAs with oncogenic or tumor-promoting effects (Oncogenic miRNAs) (Figure 2).

### Selection of miRNAs for specific functional assays

We focused mainly on HAG^low^ miRNAs, which are presumably tumor-suppressive and could provide the basis for novel lines of therapy for melanoma. miRNAs with tumor-suppressive potential were analyzed for predicted targets with TargetScan algorithm [30] and for the affected biological processes with miRanda (miRpath) algorithm [31]. All the potentially affected biological processes were ranked according to the overall P value, with a cut-off of <0.01. Further focus on the robust processes was enabled by selecting biological processes that included at least 30 candidate target genes. Remarkably, these computational steps highlighted four biological processes that are highly involved in cancer, including Wnt pathway (82 genes, P = 1.7×10^−9^), focal adhesion (100 genes, P = 8.6×10^−9^), MAPK pathway (120 genes, P = 2×10^−7^) and Phosphatidylinositol pathway (35 genes, P = 7.2×10^−3^). Twenty nine of the miRNAs were predicted to target all four processes, with only 12 having been reported to experimentally exert a suppressive effect in any cancer.

Altogether, five candidate Suppressive miRNAs, which have never been studied in melanoma, were selected for cloning and experimental tests. Three miRNAs were within expression range (miR-34a, miR-185 and miR-204, Figure 2C) and two which were silenced (miR-31 and miR-184, Figure 2B) in the HAG cells. miR-17 was selected as an example for candidate Oncogenic miRNA (Figure 2C). The differential expression of each of these miRNAs was validated in two independent RNA preparations (data not shown). Different roles of miR-17, miR-31 and miR-34a in cancer have been reported before, although never in cutaneous melanoma. In contrast, there is scarce evidence on the roles of miRNAs -184, -185 and -204 in cancer. Exemplar predicted roles in biological and molecular functions concerning these miRNAs are summarized in Supplementary Table S1 [32].

### Expression profile of selected miRNAs in clinical melanoma specimens

**Figure 3:**
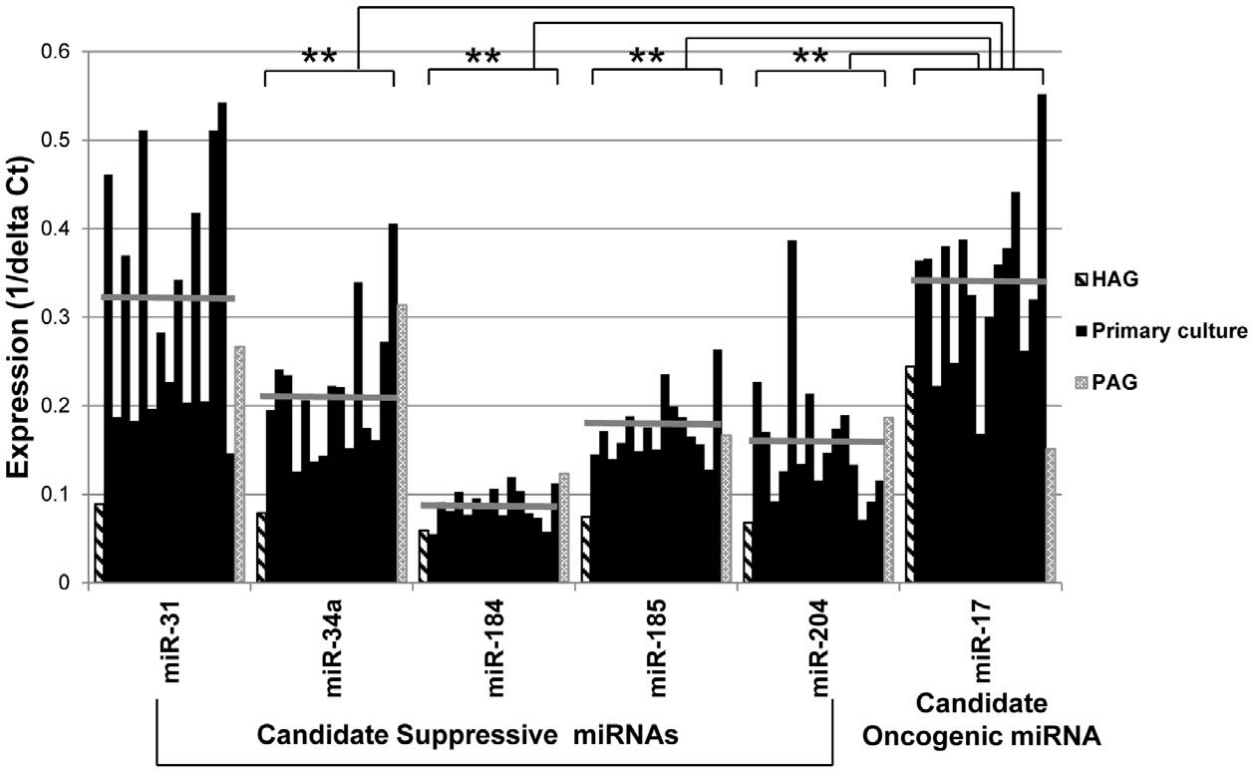
Expression analysis of selected miRNAs in clinical melanoma specimens. The expression of candidate Suppressive or Oncogenic miRNAs, as indicated, in HAG (striped bars), PAG (gray bars) and 15 low-passage primary cultures of metastatic melanoma (black bars). Horizontal line depicts the mean expression. Y-axis denotes absolute expression of each miRNA after normalization to the U6 endogenous control in each specimen, and is presented as 1/ΔCt.

Fifteen patient-derived primary cultures of melanoma were analyzed for the expression level of the selected miRNAs. All melanoma cultures were established from distant metastases. Additional data on the patients is provided in Supplementary Table S2. As expected, a considerable variability in miRNA expression was observed among the individual specimens, mainly of miR-31 (Figure 3). The mean expression level of most candidate Suppressive miRNAs in the clinical specimens was between the corresponding miRNA values in the PAG and HAG cells, except for miR-185 and miR-31. While the mean level of miR-185 was very close to the PAG cells, miR-31 levels were clearly higher even than PAG cells (Figure 3). In contrast, the mean expression of the candidate Oncogenic miR-17 among the clinical specimens was even higher than in HAG cells (Figure 3). The miRNA expression patterns in clinical specimens directly shows that most candidate Suppressive miRNAs, except for miR-31, are expressed at significantly lower levels than the candidate Oncogenic miR-17 (Figure 3). These results concur with our hypothesis and suggest that the approach used to identify functional Suppressive and Oncogenic miRNAs has physiologically-relevant grounds.

### Regulation of aggressive phenotype of melanoma cells by candidate Suppressive miRNAs

**Figure 4:**
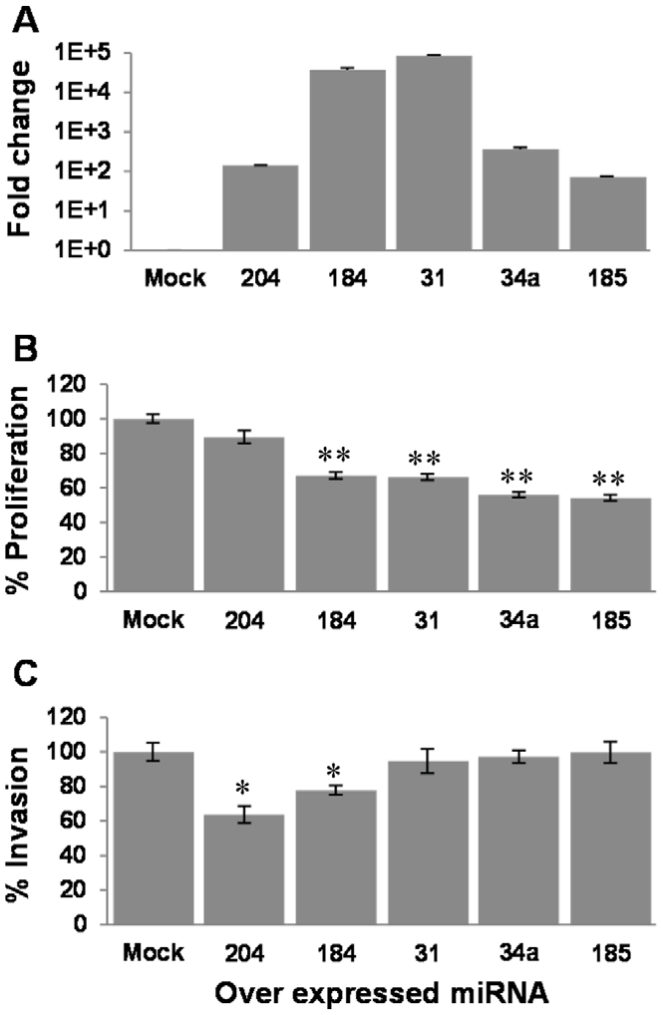
Candidate Suppressive miRNAs inhibit proliferation and invasion of melanoma cells. (A) Verification of over-expression of miRNAs in HAG transductants, as compared to mock-transduced cells. Y-axis denotes fold change above Mock-transduced cells. (B) Net proliferation of the transductants was quantified with standardized XTT test. The number of cells was determined 48 h after seeding. The number of Mock-transductants was determined as 100%. Figure shows a representative experiment out of three performed. (C) Invasion activity of transductants was quantified with 20 h-matrigel invasion assay, with correction for proliferation. The invasion rate of Mock-transduced cells was determined as 100%. Figure shows a representative experiment out of 3 performed. * denotes P<0.05, ** denotes P<0.01 (2-tailed *t-test*).

Determination of miRNAs that inhibit the aggressive phenotype of melanoma could become a basis for development of new platforms for cancer therapy. In order to evaluate the functional effect of the candidate Suppressive miRNAs on the aggressive features of melanoma, we cloned and stably over-expressed them in HAG cells to evaluate their effect in-vitro and in-vivo. An empty vector served as control. An over-expression of at least 50-fold was confirmed by real time PCR (Figure 4A). The phenotype of the transduced cells was tested *in-vitro* for proliferation, invasion and tube formation activities. Remarkably, a substantial and consistent inhibition in net proliferation was conferred by miR-31, miR-34a, miR-184 and miR-185 as compared to the control cell (Figure 4B). miR-204 did not inhibit the proliferation of HAG cells (Figure 4B). In contrast, miR-204 markedly inhibited the invasion activity of HAG cells (Figure 4C). Invasion was similarly inhibited by miR-184, but not by the other Suppressive miRNAs (Figure 4C).

**Figure 5:**
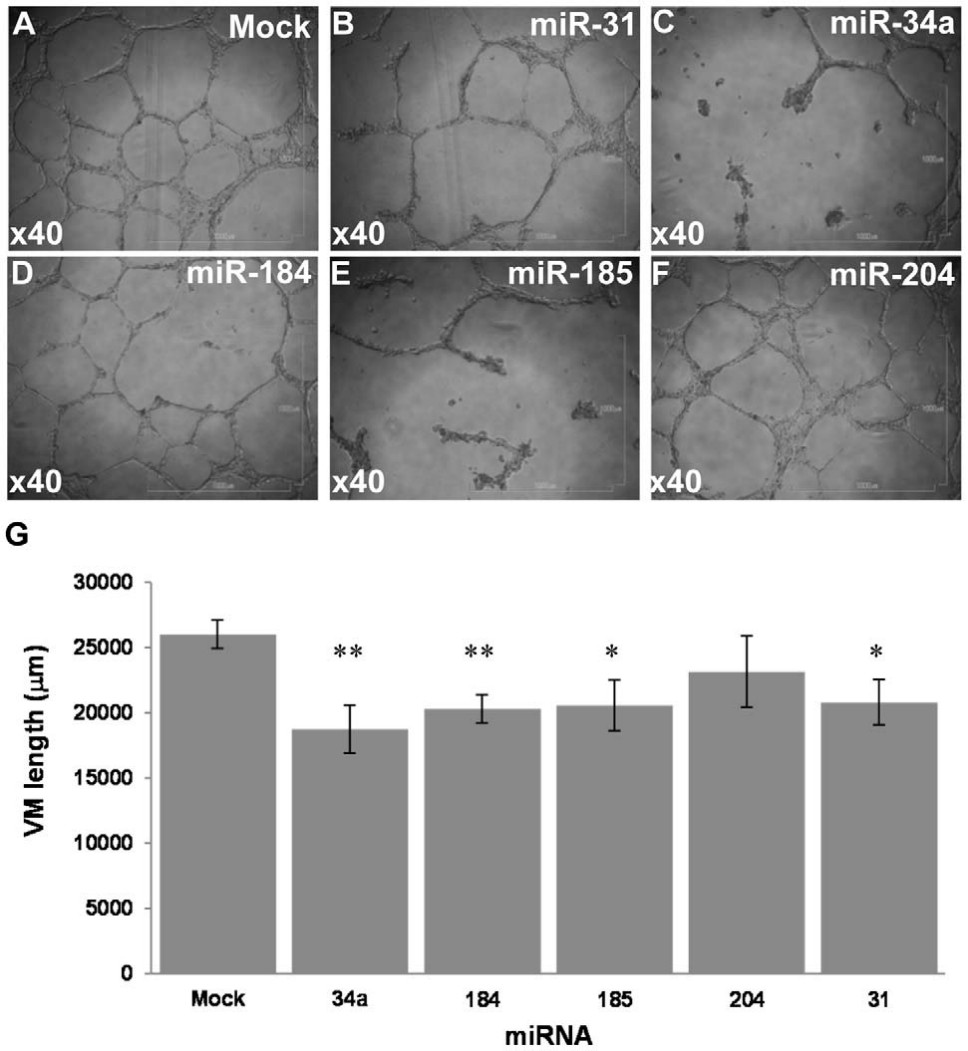
Candidate Suppressive miRNAs inhibit tube formation by melanoma cells. Tube formation ability of transductants was tested in 3D culture, 24 h after seeding. Each experiment was performed in triplicate wells. (A) A representative microphotograph of a 3D culture of Mock-transduced HAG cells; (B–F) Representative microphotographs of miRNA-transduced HAG cells, as indicated in each picture. All images were captured under ×40 magnification. All pictures (A–F) were derived from the same representative experiment shown, out of three performed. (G) Tube formation was quantified using the ImageJ analyze skeleton PlugIn. Figure shows the average length calculated for each transductant out of all microphotographs captured in all three experiments performed. * denotes P<0.05, ** denotes P<0.01 (2-tailed *t-test*).

Tube formation activity was substantially inhibited by miR-34a and miR-185, and more mildly by miR-31 and miR-184, but not by miR-204, as compared to control (Figure 5, A–F). Quantification of total tube length was performed using ImageJ. Importantly, the qualitative assessment of micrographic captures (Figure 5, A–F) concurred with the quantitative total length analysis (Figure 5G). The differential effects of the miRNAs could not be simply attributed to their differential over-expression intensities (Figure 4A). Almost all cells were viable when assayed, as evident by <5% positive trypan blue staining (data not shown).

Taken together, all five candidate Suppressive miRNAs indeed exerted significant inhibitory effects on various aggressive features of melanoma cells. This concurs with their substantial downregulation in the HAG cells (Figure 2) and their overall low expression in clinical specimens (Figure 3). This also strengthens our high-throughput miRNA screening and emphasizes its reliability.

### Facilitation of aggressive phenotype of melanoma cells by candidate Oncogenic miRNA

**Figure 6:**
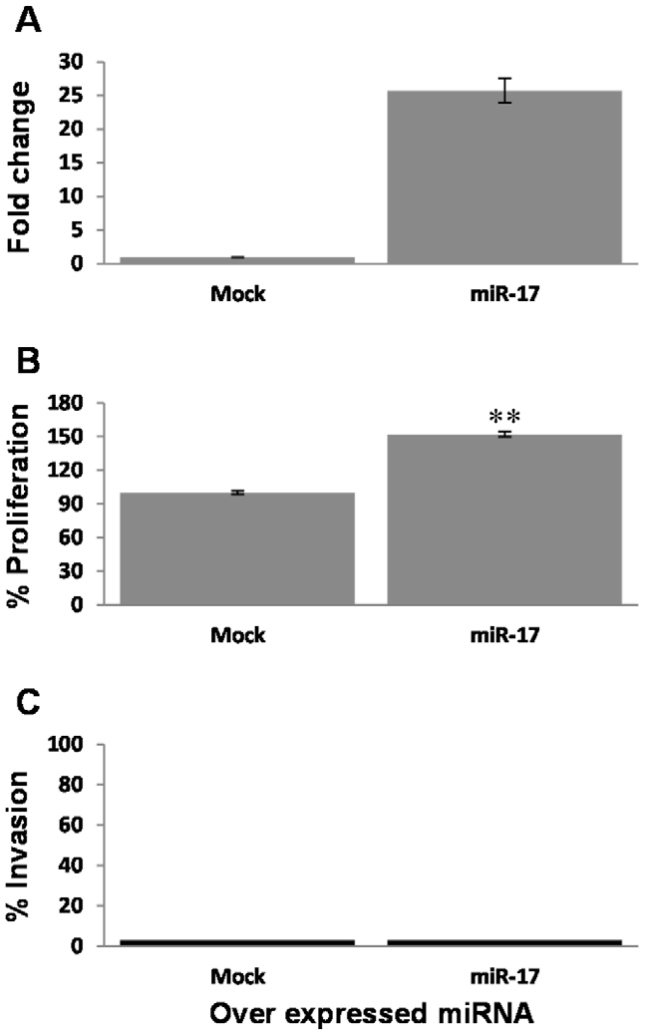
Candidate Oncogenic miRNA enhance proliferation of melanoma cells. (A) Verification of miR-17-5p over-expression in PAG transductants, as compared to mock-transduced cells. Y-axis denotes fold change above Mock-transduced cells. (B) Net proliferation of the PAG transductants was quantified with standardized XTT test. The number of cells was determined 48 h after seeding. The number of Mock-transductants was determined as 100%. Figure shows a representative experiment out of 3 performed. (C) Invasion activity of PAG transductants was quantified with 20 h-matrigel invasion assay, with correction for proliferation. Y-axis denotes the percentage of invaded cells. Figure shows a representative experiment out of three performed. ** denotes P<0.01 (2-tailed *t-test*).

Since the miRNA 17–92 clusters' functional role in cancer is well established, yet never has it been tested in cutaneous melanoma, we evaluated miR-17 for its effect on the aggressiveness of PAG cells. miR-17 was cloned and stably over-expressed in the PAG cells. An empty vector served as control. A 25-fold over-expression of miR-17 was verified by real time PCR (Figure 6A). Importantly, miR-17-transduced PAG cells displayed a significantly enhanced proliferative activity (Figure 6B) but not invasive ability (Figure 6C) or tube formation activity (data not shown). These results support the potentially oncogenic effects of miR-17 in melanoma.

### miRNA-mediated regulation of aggressive melanoma features *in-vivo*

**Figure 7:**
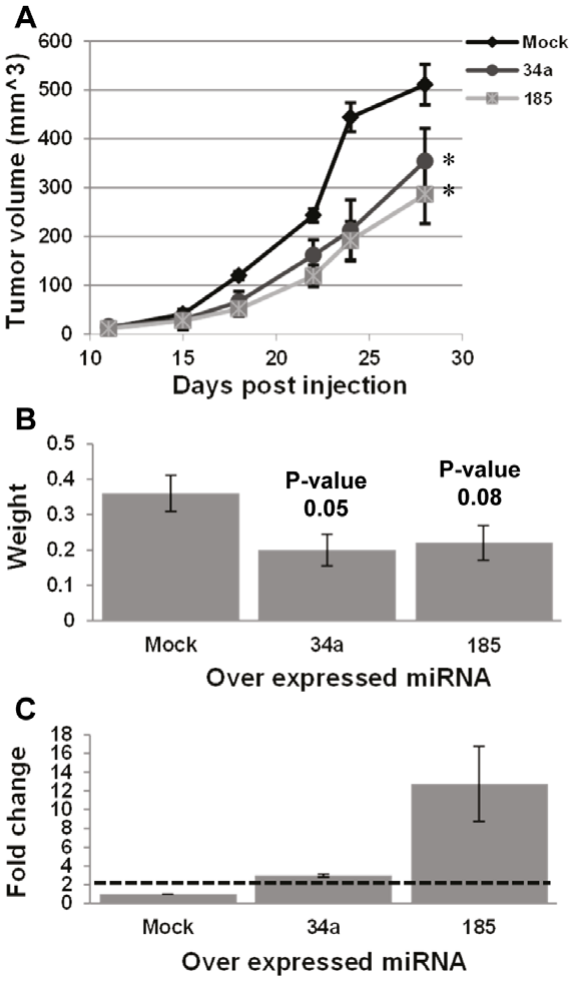
Suppressive miRNAs inhibit tumor growth *in-vivo*. (A) Monitoring of tumor growth in SCID-NOD mice. Each group was comprised of seven mice. A representative experiment out of four performed is shown. Statistical significance was tested with 2-tailed-paired *t-test*; (B) Mean weight of tumors explanted upon termination of the experiment. Statistical significance was tested with 2-tailed *t-test*; (C) Over-expression of transduced miRNAs was confirmed upon termination of the experiment in all tumors with qPCR. * denotes P<0.05.

In cancer biology, *in-vitro* results often do not necessarily reflect *in-vivo* behavior. To this end, the effects of selected Suppressive miRNAs were further assessed *in-vivo* in melanoma xenograft models. The effect of the Suppressive miRNAs, miR-34a and miR-185 on tumor growth was measured following subcutaneous injection of 3×10^5^ HAG transductants. Tumor masses were monitored for 28 days post injection. Over-expression of specific miRNAs was confirmed pre-injection (data not shown). Importantly, a statistically significant inhibition in tumor growth was observed in both miR-34a and miR-185 transducatnts, as compared to control tumors (Figure 7A). Concurring with these results, *ex-vivo* weighing of tumor explants upon termination of the experiments confirmed that the average tumor mass of both miR-34a and miR-185 transducatnts was lower than Mock-transduced tumors (Figure 7B). The *in-vivo* over-expression of the transduced miRNAs was confirmed in the tumor explants (Figure 7C). These results corroborate with the expression results and functional suppressive effects demonstrated *in-vitro* (Figure 4–5).

## Discussion

Up to date, most attempts to characterize the roles of miRNAs in cancer in general or melanoma in particular, have focused on differential profiling of normal cells compared to their malignant counterpart cells [19], [20]. This approach enabled the identification of miRNAs that participate in the process of malignant transformation. Here we focused on identification of miRNAs that directly regulate cancer aggressive features of melanoma cells. The objective of this approach was to map the miRNAs that could be involved mechanistically in different disease phenotypes and eventually delineate their regulatory role of aggressive cancer features. We used two isogenic matched melanoma cell lines that were derived of two different metastases from the same patient. These cell lines significantly differ in their invasive, proliferative and tumorigenic properties (Figure 1). Due to the shared genetic background of the cells, our rationale was that the differential expression of miRNAs would correlate well with their differential aggressive phenotype.

High-throughput screening revealed a large cohort of differentially expressed miRNAs between highly (HAG) and poorly (PAG)-aggressive melanoma cell-lines (Figure 2). We hypothesized that HAG^low^ miRNAs are suppressive and that HAG^high^ miRNAs are oncogenic. Indeed, we provide substantial evidence supporting our hypothesis, by ectopic expression of five selected HAG^low^ miRNAs in HAG cells (Figures 4, 5, 7) and one HAG^high^ miRNA in PAG cells, as a representative of this group (Figure 6). This is the first report to successfully demonstrate a systematic approach for a methodological identification of miRNAs that directly regulate aggressive cancer features. The wealth of identified miRNAs that regulate features of melanoma cell aggressiveness supports the use of isogenic cell lines with differential phenotype as a screening platform.

The expression of these miRNAs was validated in 15 low-passage primary cultures, with the mean level of miR-17 being higher than that of the Suppressive miRs, thus confirming the physiological relevance (Figure 3). The expression of these miRNAs should be further studied in the future in progression tissue microarrays, and their prognostic value tested accordingly.

In the present work we focused on a group of either well characterized miRNAs known to have a role in other malignancies (miR-17, miR-31 and miR-34a), and on a second group of relatively unstudied miRNAs with regard to their role in malignant processes (miR-184, miR-185, miR-204). Noteworthy, the roles of all of these miRNAs have never been described in cutaneous melanoma.

miR-31 was reported to exert inhibitory effects on metastasis in breast cancer [33], [34] and in mesothelioma [17], while it has a potential oncogenic role in as head and neck squamous cell carcinoma and lung cancer [35], [36]. miR-34a was consistently reported as a suppressive miRNA in many malignancies [37]. Our results concur with the suggested inhibitory role for miR-34a and miR-31. Moreover, miR-34a has been previously reported to target c-Met [16]. Since c-Met has been reported to participate in tubulogenesis processes through the c-Met/HGF system [38], it is tempting to speculate that the mechanism by which miR-34a inhibits tube formation is via this gene. The oncogenic properties of miR-17-5p were discussed in earlier publications in other malignancies [13]. In agreement with these reports, ectopic expression of miR-17-5p in the PAG cells enhanced proliferation rate (Figure 6B).

In contrast, very little is known about miR-184, miR-185 and miR-204 in cancer. Most current studies on miR-184 and miR-204 focus on their roles in development and morphogenesis, which could be predicted computationally (Supplementary Table S1). The few publications concerning miR-184 in cancer showed contradictory effects, either suppressive [39] or oncogenic [40]. A single report showed that miR-204 inhibited metastasis in head and neck squamous cell carcinoma [41]. Another report described downregulation of miR-204 in highly invasive melanoma sub-line compared to its non-invasive isogenic counterpart [19], supporting our findings regarding the invasion-inhibiting activity of miR-204 (Fig. 4C). miR-185 is still mostly unstudied, but recently it was demonstrated to exert suppressive effects in several malignancies through targeting Six1 oncogene [42], [43]. The future challenge would be to delineate the target gene networks of these miRNAs and understand the molecular basis for their tumor-suppressive role.

The effects of each individual miRNA may not apparently be identical between *in-vitro* and *in-vivo* setups. For example, while miRNAs-34a and -185 exhibited strong anti-proliferative effects *in-vitro* (Figure 4B), they affected much more moderately tumor growth rate *in-vivo* (Figure 7A). This could be attributed to a major difference in expression intensity between *in-vitro* and *in-vivo* settings (Figure 4A and 7C). Nevertheless, the *in-vivo* experiments suggest that even when the over-expression level is relatively weak (Figure 7C), an experimental outcome that takes into account several biological processes, such as tumor growth, can still reflect the suppressive effect of the over-expressed miRNA (Figure 7). Moreover, these experiments simplify the possibility of combinatorial regulatory effects of different miRNAs, which can work either in concert or interference [44]. Thus, a coordinated downregulation and upregulation of miRNAs may shape a certain phenotype even when the alterations in each miRNA expression level are not extreme. Indeed, we have observed several direct and inverse statistical correlations between the tested Suppressive miRNAs in the clinical melanoma specimens, which could suggest synergistic or antagonistic effects among them (data not shown). The outcome of the interactions between the different Suppressive miRNAs should be further investigated to deepen our understanding of phenotype shaping by combinatorial miRNA patterns, and since synergistic inhibitory combinations could provide a solid lead for innovative therapy.

In conclusion, this is the first report that successfully demonstrated a systematic approach for identification of miRNAs that directly regulate aggressive cancer features. We described six miRNAs contributing to the aggressive phenotype of cutaneous melanoma both *in-vitro* and *in-vivo*, and linked these observations to clinical samples. Identification of miRNAs that shape the aggressive phenotype of the disease may lead to the development of innovative future therapy and molecular staging systems. Recent publications showed the possibility of targeting experimental melanoma metastases using nanoparticles carrying miR-34a [45] or a synthetic miRNA agonist [46].

## Materials and Methods

### Ethics Statement

All animal work in mice was performed following approval of an Institutional Review Board of Sheba Medical Center (562/2010). All clinical samples derived from patients, from which melanoma cultures were establish, were obtained following approval of the ethical committee of the Israel Ministry of Health (IMoH approval No. 3518/2004). All patients have willingly signed on an informed consent form.

### Cells

The two isogenic human cutaneous melanoma cell lines C8161 cells (Highly aggressive - HAG) and the poorly aggressive C81-61 (Poorly aggressive - PAG) [29] were kindly provided by Dr Mary Hendrix (Children's Memorial Research Center, Chicago, USA). These cell lines were derived of two different metastases from the same patient. Primary melanoma cultures were developed from surgically removed metastatic melanoma lesions (IMoH approval No. 3518/2004) and were grown as previously described [47]. PAG cells were grown in Ham's F10 medium supplement with 15% FBS, Pen/Strep and 1× MITO + (BD Biosciences). Normal Human Epidermal Melanocytes were maintained in a serum free unique medium (PromoCell). 293T cells (ATCC) were maintained in DMEM (Gibco/Invitrogen) containing 10% FBS (DMEM/FBS).

### miRNA expression analysis

Total RNA was isolated using the mirVana miRNA Isolation Kit (Ambion). High-throughput screening of miRNA was performed by real time PCR using TLDA format TaqMan MicroRNA kits (Applied Biosystems). Expression of specific miRNAs and U6 RNA (as endogenous control) was performed following reverse transcription from <200 nt RNA fractions, using the TaqMan MicroRNA kits (Applied Biosystems). Cutoff levels for significance were determined as at least 4-fold ratio between tested samples and cycle 36 as the limit for expression range, based on our previous evaluation of this technology. Quantification of miRNAs expression in xenografts was performed following tumor excision, homogenization (Polytron homogenizer) in Tri reagent (Sigma) and RNA purification with Trizol.

### Cloning of miRNAs into mammalian expression vector

Genomic DNA was extracted from cells with the Wizard Genomic DNA Purification Kit (Promega). miRNAs were amplified with PCR from genomic DNA using specific primers (Supplementary Table S3). Each amplified miRNA included the flanking genomic sequences of 110 bp from both sides. The amplicon was cloned into the pQCXIP vector (CloneTech) using the NotI and EcoRI restriction enzymes (New England BioLabs). Empty pQCXIP served as negative control. All cloned constructs were fully sequenced.

### Cell transductions

2×10^5^ 293T cells were seeded in a 6-well plate and cultured overnight in DMEM (Gibco/Invitrogen) containing 10% FBS (DMEM/FBS). On day 1, cells were transfected with a mixture of 1 µg GAG-POL, 1 µg Envelope, 2 µg of each of the pQCXIP constructs and 6 µl of Turbofect reagent (Fermentas). After six hours of incubation at 37°C, the cells were washed and re-cultured in fresh DMEM/FBS. On day 2, 5×10^4^ melanoma cells were placed in each well of 6-well plates and cultured overnight in DMEM/FBS. On day 3, the melanoma cells were infected with 6 ml of 0.45 µm-filtered virion-containing medium of the 293T cells. After incubation at 37°C for 6 hours, the infected melanoma cells were washed and re-cultured with fresh DMEM./FBS. The aforementioned infection procedure was repeated the next day on the same melanoma culture. 48 hours after the second infection, selection was performed by addition of 1.2 µg/ml puromycine into culture medium.

### Net cell proliferation

Melanoma cells (3×10^3^) were seeded in triplicate wells in 96F-well microplates. Net proliferation was determined by XTT colorimetric assay (Biological-Industries), according to manufacturer's instruction. Following background subtraction, the O.D. values were transformed into viable cells counts according to the specific regression equation that was determined for each cell line tested.

### Invasion assay

Melanoma cells (2×10^5^) were seeded in the upper wells of Transwell invasion system on Matrigel (BD) coated ThinCerts PET 8-µm membranes (Greiner-bio-one) in RPMI 1640 supplemented with 0.1% FBS. The lower well contained the same medium with 10% FBS. After 24 hours of incubation in humidified 5% CO_2_ incubator, the upper well content, which contained non-invading cells, was removed using cotton swabs. The amount of cells that invaded through the membranes was measured by standardized XTT staining (as above) and corrected for proliferation. Percent of invasion was calculated out of the number of cells seeded.

### Tube formation assay and image analysis

Tube formation activity *in-vitro* on Matrigel (BD) was determined in 96-well microplates as previously described [48]. Tube formation was quantified after 24 h by an image analysis process using whole field image capture (×40 microscopic images) to avoid any bias. In principle, we quantified the distribution of the network lengths. First, a threshold was manually set to specifically demonstrate the network structures in the image. The quality and resolution of the images allowed reliable and exclusive threshold of the networks without the need of image filtering. Images were then placed in bins and subjected to the “Skeletonize” function of ImageJ software. The corresponding lengths was measured using the 2D/3D skeleton PlugIn [49] for the NIH ImageJ software [50].

### Human melanoma xenograft model in SCID-NOD mice

3×10^5^ miRNA transfected HAG cells were injected subcutaneously to the thigh of 8w old SCID-NOD mice to create human melanoma xenografts. Experimental groups included control cells and miRNA over-expressing cells. Each group included at least 6 mice. Mice were monitored 3 times per week for tumor volume by caliper measurement. Tumor volume was calculated as (small diameter)^2^×(large diameter)/2. At termination of each experiment, tumor masses were extracted, weighed, photographed and snap frozen in liquid nitrogen. All animal work was performed following approval of an Institutional Review Board of Sheba Medical Center (562/2010).

## Supporting Information

Table S1 Summary of predicted information for indicated miRNAs, obtained from ToppGene [32].
(DOC)

Table S2 Clinical data of all primary melanoma cultures derived from patients.
(DOC)

Table S3 List of primers used to clone the miRNAs examined in this study.
(DOC)

## References

[1] Gloster HM, Brodland DG (1996). The epidemiology of skin cancer.. Dermatol Surg.

[2] Melnikova VO, Bar-Eli M (2008). Transcriptional control of the melanoma malignant phenotype.. Cancer Biol Ther.

[3] Bennett DC (2008). How to make a melanoma: what do we know of the primary clonal events?. Pigment Cell Melanoma Res.

[4] Hendrix MJ, Seftor EA, Hess AR, Seftor RE (2003). Molecular plasticity of human melanoma cells.. Oncogene.

[5] Frenkel S, Barzel I, Levy J, Lin AY, Bartsch DU (2008). Demonstrating circulation in vasculogenic mimicry patterns of uveal melanoma by confocal indocyanine green angiography.. Eye (Lond).

[6] Sun B, Zhang S, Zhang D, Du J, Guo H (2006).

[7] Li M, Gu Y, Zhang Z, Zhang S, Zhang D (2010). Vasculogenic mimicry: a new prognostic sign of gastric adenocarcinoma.. Pathol Oncol Res.

[8] Tap WD, Gong KW, Dering J, Tseng Y, Ginther C (2010). Pharmacodynamic characterization of the efficacy signals due to selective BRAF inhibition with PLX4032 in malignant melanoma.. Neoplasia.

[9] Bartel DP (2009). MicroRNAs: target recognition and regulatory functions.. Cell.

[10] Ma L, Weinberg RA (2008). MicroRNAs in malignant progression.. Cell Cycle.

[11] Griffiths-Jones S, Saini HK, van Dongen S, Enright AJ (2008). miRBase: tools for microRNA genomics.. Nucleic Acids Res.

[12] Friedman RC, Farh KK, Burge CB, Bartel DP (2009). Most mammalian mRNAs are conserved targets of microRNAs.. Genome Res.

[13] Garzon R, Calin GA, Croce CM (2009). MicroRNAs in Cancer.. Annu Rev Med.

[14] Calin GA, Croce CM (2006). MicroRNA signatures in human cancers.. Nat Rev Cancer.

[15] Klein U, Lia M, Crespo M, Siegel R, Shen Q (2010). The DLEU2/miR-15a/16-1 cluster controls B cell proliferation and its deletion leads to chronic lymphocytic leukemia.. Cancer Cell.

[16] Yan D, Zhou X, Chen X, Hu DN, Dong XD (2009). MicroRNA-34a inhibits uveal melanoma cell proliferation and migration through downregulation of c-Met.. Invest Ophthalmol Vis Sci.

[17] Ivanov SV, Goparaju CM, Lopez P, Zavadil J, Toren-Haritan G (2010). Pro-tumorigenic effects of miR-31 loss in mesothelioma.. J Biol Chem.

[18] Mu P, Han YC, Betel D, Yao E, Squatrito M (2009). Genetic dissection of the miR-17∼92 cluster of microRNAs in Myc-induced B-cell lymphomas.. Genes Dev.

[19] Mueller DW, Rehli M, Bosserhoff AK (2009). miRNA expression profiling in melanocytes and melanoma cell lines reveals miRNAs associated with formation and progression of malignant melanoma.. J Invest Dermatol.

[20] Caramuta S, Egyhazi S, Rodolfo M, Witten D, Hansson J (2010). MicroRNA expression profiles associated with mutational status and survival in malignant melanoma.. J Invest Dermatol.

[21] Jukic DM, Rao UN, Kelly L, Skaf JS, Drogowski LM (2010). Microrna profiling analysis of differences between the melanoma of young adults and older adults.. J Transl Med.

[22] Segura MF, Belitskaya-Levy I, Rose AE, Zakrzewski J, Gaziel A (2010). Melanoma MicroRNA signature predicts post-recurrence survival.. Clin Cancer Res.

[23] Chen J, Feilotter HE, Pare GC, Zhang X, Pemberton JG (2010). MicroRNA-193b represses cell proliferation and regulates cyclin D1 in melanoma.. Am J Pathol.

[24] Muller DW, Bosserhoff AK (2008). Integrin beta 3 expression is regulated by let-7a miRNA in malignant melanoma.. Oncogene.

[25] Levy C, Khaled M, Iliopoulos D, Janas MM, Schubert S (2010). Intronic miR-211 assumes the tumor suppressive function of its host gene in melanoma.. Mol Cell.

[26] Mazar J, DeYoung K, Khaitan D, Meister E, Almodovar A (2010). The regulation of miRNA-211 expression and its role in melanoma cell invasiveness.. PLoS One.

[27] Segura MF, Hanniford D, Menendez S, Reavie L, Zou X (2009). Aberrant miR-182 expression promotes melanoma metastasis by repressing FOXO3 and microphthalmia-associated transcription factor.. Proc Natl Acad Sci U S A.

[28] Felicetti F, Errico MC, Bottero L, Segnalini P, Stoppacciaro A (2008). The promyelocytic leukemia zinc finger-microRNA-221/-222 pathway controls melanoma progression through multiple oncogenic mechanisms.. Cancer Res.

[29] Welch DR, Bisi JE, Miller BE, Conaway D, Seftor EA (1991). Characterization of a highly invasive and spontaneously metastatic human malignant melanoma cell line.. Int J Cancer.

[30] Lewis BP, Burge CB, Bartel DP (2005). Conserved seed pairing, often flanked by adenosines, indicates that thousands of human genes are microRNA targets.. Cell.

[31] Maragkakis M, Reczko M, Simossis VA, Alexiou P, Papadopoulos GL (2009). DIANA-microT web server: elucidating microRNA functions through target prediction.. Nucleic Acids Res.

[32] Chen J, Bardes EE, Aronow BJ, Jegga AG (2009). ToppGene Suite for gene list enrichment analysis and candidate gene prioritization.. Nucleic Acids Res.

[33] Valastyan S, Reinhardt F, Benaich N, Calogrias D, Szasz AM (2009). A pleiotropically acting microRNA, miR-31, inhibits breast cancer metastasis.. Cell.

[34] Valastyan S, Chang A, Benaich N, Reinhardt F, Weinberg RA (2010). Concurrent suppression of integrin alpha5, radixin, and RhoA phenocopies the effects of miR-31 on metastasis.. Cancer Res.

[35] Liu X, Sempere LF, Ouyang H, Memoli VA, Andrew AS (2010). MicroRNA-31 functions as an oncogenic microRNA in mouse and human lung cancer cells by repressing specific tumor suppressors.. J Clin Invest.

[36] Liu CJ, Tsai MM, Hung PS, Kao SY, Liu TY (2010). miR-31 ablates expression of the HIF regulatory factor FIH to activate the HIF pathway in head and neck carcinoma.. Cancer Res.

[37] Hermeking H (2010). The miR-34 family in cancer and apoptosis.. Cell Death Differ.

[38] Pan BS, Chan GK, Chenard M, Chi A, Davis LJ (2010). MK-2461, a novel multitargeted kinase inhibitor, preferentially inhibits the activated c-Met receptor.. Cancer Res.

[39] Foley NH, Bray IM, Tivnan A, Bryan K, Murphy DM (2010). MicroRNA-184 inhibits neuroblastoma cell survival through targeting the serine/threonine kinase AKT2.. Mol Cancer.

[40] Wong TS, Liu XB, Wong BY, Ng RW, Yuen AP (2008). Mature miR-184 as Potential Oncogenic microRNA of Squamous Cell Carcinoma of Tongue.. Clin Cancer Res.

[41] Lee Y, Yang X, Huang Y, Fan H, Zhang Q (2010). Network modeling identifies molecular functions targeted by miR-204 to suppress head and neck tumor metastasis.. PLoS Comput Biol.

[42] Imam JS, Buddavarapu K, Lee-Chang JS, Ganapathy S, Camosy C (2010). MicroRNA-185 suppresses tumor growth and progression by targeting the Six1 oncogene in human cancers.. Oncogene.

[43] Takahashi Y, Forrest AR, Maeno E, Hashimoto T, Daub CO (2009). MiR-107 and MiR-185 can induce cell cycle arrest in human non small cell lung cancer cell lines.. PLoS One.

[44] Nachmani D, Lankry D, Wolf DG, Mandelboim O (2010). The human cytomegalovirus microRNA miR-UL112 acts synergistically with a cellular microRNA to escape immune elimination.. Nat Immunol.

[45] Chen Y, Zhu X, Zhang X, Liu B, Huang L (2010). Nanoparticles Modified With Tumor-targeting scFv Deliver siRNA and miRNA for Cancer Therapy.. Mol Ther.

[46] Wiggins JF, Ruffino L, Kelnar K, Omotola M, Patrawala L (2010). Development of a lung cancer therapeutic based on the tumor suppressor microRNA-34.. Cancer Res.

[47] Besser MJ, Shapira-Frommer R, Treves AJ, Zippel D, Itzhaki O (2010). Clinical responses in a phase II study using adoptive transfer of short-term cultured tumor infiltration lymphocytes in metastatic melanoma patients.. Clin Cancer Res.

[48] Maniotis AJ, Folberg R, Hess A, Seftor EA, Gardner LMG (1999). Vascular Channel Formation by Human Melanoma Cells in Vivo and in Vitro: Vasculogenic Mimicry.. Am J Pathol.

[49] Arganda-Carreras I, Fernandez-Gonzalez R, Munoz-Barrutia A, Ortiz-De-Solorzano C (2010). 3D reconstruction of histological sections: Application to mammary gland tissue.. Microsc Res Tech.

[50] Abramoff MD, Magelhaes PJ, Ram SJ (2004). Image Processing with ImageJ.. Biophotonics International.

