# Supplementary material for: Regulation of Cancer Aggressive Features in Melanoma Cells by MicroRNAs
Source: PLoS One. 2011 Apr 25;6(4):e18936. doi: 10.1371/journal.pone.0018936 (PMC3081841; doi:10.1371/journal.pone.0018936)
Supplement: Table S1 — Summary of predicted information for indicated miRNAs, obtained from ToppGene [32]. (DOC) [file pone.0018936.s001.doc]

**Supplementary Table S1**

| **HSA-mir-34a** | | | | **HSA -mir-17** | | | | **HSA-mir-31** | |
| --- | --- | --- | --- | --- | --- | --- | --- | --- | --- |
| **Type** | **P value** | **Targets** | **Effect** | **Type** | **P value** | **Targets** | **Effect** | **Effect** | |
| **Molecular Function** | | | **Suppr** | **Molecular Function** | | | **Onco** | **Onco** | **Suppr** |
| Protein domain specific binding | 0.05 | 27/440 | [1,2,3,4,5,6,7,8,9,10,11,12,13,14,15,16,17,18,19,20,21,22,23,24,25,26] | Protein kinase activity | <0.01 | 74/617 | [27,28,29,30,31,32,33,34,35,36] | [37,38] | [39,40,41,42,43] |
|  |  |  |  | Phosphotrans-ferase activity | <0.01 | 82/731 |  |  |  |
|  |  |  |  | Transcription factor activity | <0.01 | 102/1014 |  |  |  |
| **Biological Process** | | |  | **Biological Process** | | |  |  |  |
| Cell projection morphogenesis | <0.01 | 40/540 |  | Protein amino acid phosphorylation | <0.01 | 99/838 |  |  |  |
| Regulation of cell differentiation | <0.01 | 39/636 |  | Protein localization | <0.01 | 119/1110 |  |  |  |
| Positive regulation of developmental process | <0.01 | 48/867 |  | Negative regulation of gene expression | 0.02 | 65/614 |  |  |  |
| Cell morphogenesis | 0.04 | 48/930 |  | **Pathway** | | |  |  |  |
|  |  |  |  | Pathways in cancer | 0.02 | 41/331 |  |  |  |
|  |  |  |  | BMP receptor signaling | 0.05 | 11/42 |  |  |  |

| **HSA-mir-184** | | | | | **HSA-mir-185** | | | | **HSA-mir-204** | | | |
| --- | --- | --- | --- | --- | --- | --- | --- | --- | --- | --- | --- | --- |
| **Type** | **P value** | **Targets** | **Effect** | | **Type** | **P value** | **Targets** | **Effect** | **Type** | **P value** | **Targets** | **Effect** |
| **Molecular Function** | | | **Onco** | **Suppr** | **Molecular Function** | | | **Suppr** | **Molecular Function** | | | **Suppr** |
| Extracellular matrix binding | 0.02 | 3/39 | [44,45] | [46] | Sequence-specific DNA binding | <0.01 | 60/665 | [47,48] | Transmembrane receptor protein kinase activity | <0.01 | 20/82 | [49] |
|  |  |  |  |  | Transcription factor activity | <0.01 | 80/1014 |  | Protein kinase activity | <0.01 | 60/617 |  |
| **Biological Process** | | |  |  | **Biological Process** | | |  | **Biological Process** | | |  |
| Embryonic development | 0.01 | 10/738 |  |  | Embryonic development | <0.01 | 70/738 |  | Differentiation related cell morphogenesis | <0.01 | 47/373 |  |
|  |  |  |  |  | Differentiation related cell morphogenesis | <0.01 | 43/373 |  | Cell development | <0.01 | 99/1111 |  |
|  |  |  |  |  | Cell development | <0.01 | 90/1111 |  |  |  |  |  |
| Blood vessel development | 0.03 | 7/364 |  |  | Regulation of cell differentiation | <0.01 | 56/636 |  | Cell projection morphogenesis | <0.01 | 57/540 |  |
|  |  |  |  |  | **Pathway** | | |  | **Pathway** | | |  |
| Vasculature development | 0.04 | 7/372 |  |  | Actions of Nitric Oxide in the Heart | 0.01 | 7/18 |  | Axon guidance | 0.002 | 22/130 |  |
|  |  |  |  |  |  |  |  |  | Protein kinase A (PKA) signaling | 0.01 | 12/47 |  |

**References**

1. Chang TC, Wentzel EA, Kent OA, Ramachandran K, Mullendore M, et al. (2007) Transactivation of miR-34a by p53 broadly influences gene expression and promotes apoptosis. Mol Cell 26: 745-752.

2. Chim CS, Wong KY, Qi Y, Loong F, Lam WL, et al. (2010) Epigenetic inactivation of the miR-34a in hematological malignancies. Carcinogenesis 31: 745-750.

3. Christoffersen NR, Shalgi R, Frankel LB, Leucci E, Lees M, et al. (2010) p53-independent upregulation of miR-34a during oncogene-induced senescence represses MYC. Cell Death Differ 17: 236-245.

4. Cole KA, Attiyeh EF, Mosse YP, Laquaglia MJ, Diskin SJ, et al. (2008) A functional screen identifies miR-34a as a candidate neuroblastoma tumor suppressor gene. Mol Cancer Res 6: 735-742.

5. Corney DC, Hwang CI, Matoso A, Vogt M, Flesken-Nikitin A, et al. (2010) Frequent downregulation of miR-34 family in human ovarian cancers. Clin Cancer Res 16: 1119-1128.

6. Dalgard CL, Gonzalez M, deNiro JE, O'Brien JM (2009) Differential microRNA-34a expression and tumor suppressor function in retinoblastoma cells. Invest Ophthalmol Vis Sci 50: 4542-4551.

7. Fujita Y, Kojima K, Hamada N, Ohhashi R, Akao Y, et al. (2008) Effects of miR-34a on cell growth and chemoresistance in prostate cancer PC3 cells. Biochem Biophys Res Commun 377: 114-119.

8. Guessous F, Zhang Y, Kofman A, Catania A, Li Y, et al. (2010) microRNA-34a is tumor suppressive in brain tumors and glioma stem cells. Cell Cycle 9.

9. Hermeking H (2010) The miR-34 family in cancer and apoptosis. Cell Death Differ 17: 193-199.

10. Kumamoto K, Spillare EA, Fujita K, Horikawa I, Yamashita T, et al. (2008) Nutlin-3a activates p53 to both down-regulate inhibitor of growth 2 and up-regulate mir-34a, mir-34b, and mir-34c expression, and induce senescence. Cancer Res 68: 3193-3203.

11. Li N, Fu H, Tie Y, Hu Z, Kong W, et al. (2009) miR-34a inhibits migration and invasion by down-regulation of c-Met expression in human hepatocellular carcinoma cells. Cancer Lett 275: 44-53.

12. Li Y, Guessous F, Zhang Y, Dipierro C, Kefas B, et al. (2009) MicroRNA-34a inhibits glioblastoma growth by targeting multiple oncogenes. Cancer Res 69: 7569-7576.

13. Lodygin D, Tarasov V, Epanchintsev A, Berking C, Knyazeva T, et al. (2008) Inactivation of miR-34a by aberrant CpG methylation in multiple types of cancer. Cell Cycle 7: 2591-2600.

14. Pang RT, Leung CO, Ye TM, Liu W, Chiu PC, et al. (2010) MicroRNA-34a suppresses invasion through downregulation of Notch1 and Jagged1 in cervical carcinoma and choriocarcinoma cells. Carcinogenesis 31: 1037-1044.

15. Merkel O, Asslaber D, Pinon JD, Egle A, Greil R (2010) Interdependent regulation of p53 and miR-34a in chronic lymphocytic leukemia. Cell Cycle 9: 2764-2768.

16. Pang RT, Leung CO, Ye TM, Liu W, Chiu PC, et al. MicroRNA-34a suppresses invasion through downregulation of Notch1 and Jagged1 in cervical carcinoma and choriocarcinoma cells. Carcinogenesis 31: 1037-1044.

17. Rokhlin OW, Scheinker VS, Taghiyev AF, Bumcrot D, Glover RA, et al. (2008) MicroRNA-34 mediates AR-dependent p53-induced apoptosis in prostate cancer. Cancer Biol Ther 7: 1288-1296.

18. Sun F, Fu H, Liu Q, Tie Y, Zhu J, et al. (2008) Downregulation of CCND1 and CDK6 by miR-34a induces cell cycle arrest. FEBS Lett 582: 1564-1568.

19. Tarasov V, Jung P, Verdoodt B, Lodygin D, Epanchintsev A, et al. (2007) Differential regulation of microRNAs by p53 revealed by massively parallel sequencing: miR-34a is a p53 target that induces apoptosis and G1-arrest. Cell Cycle 6: 1586-1593.

20. Tazawa H, Tsuchiya N, Izumiya M, Nakagama H (2007) Tumor-suppressive miR-34a induces senescence-like growth arrest through modulation of the E2F pathway in human colon cancer cells. Proc Natl Acad Sci U S A 104: 15472-15477.

21. Wei JS, Song YK, Durinck S, Chen QR, Cheuk AT, et al. (2008) The MYCN oncogene is a direct target of miR-34a. Oncogene 27: 5204-5213.

22. Welch C, Chen Y, Stallings RL (2007) MicroRNA-34a functions as a potential tumor suppressor by inducing apoptosis in neuroblastoma cells. Oncogene 26: 5017-5022.

23. Wiggins JF, Ruffino L, Kelnar K, Omotola M, Patrawala L, et al. (2010) Development of a lung cancer therapeutic based on the tumor suppressor microRNA-34. Cancer Res 70: 5923-5930.

24. Yamakuchi M, Ferlito M, Lowenstein CJ (2008) miR-34a repression of SIRT1 regulates apoptosis. Proc Natl Acad Sci U S A 105: 13421-13426.

25. Yamakuchi M, Lowenstein CJ (2009) MiR-34, SIRT1 and p53: the feedback loop. Cell Cycle 8: 712-715.

26. Yan D, Zhou X, Chen X, Hu DN, Dong XD, et al. (2009) MicroRNA-34a inhibits uveal melanoma cell proliferation and migration through downregulation of c-Met. Invest Ophthalmol Vis Sci 50: 1559-1565.

27. Cloonan N, Brown MK, Steptoe AL, Wani S, Chan WL, et al. (2008) The miR-17-5p microRNA is a key regulator of the G1/S phase cell cycle transition. Genome Biol 9: R127.

28. Connolly E, Melegari M, Landgraf P, Tchaikovskaya T, Tennant BC, et al. (2008) Elevated expression of the miR-17-92 polycistron and miR-21 in hepadnavirus-associated hepatocellular carcinoma contributes to the malignant phenotype. Am J Pathol 173: 856-864.

29. Fontana L, Fiori ME, Albini S, Cifaldi L, Giovinazzi S, et al. (2008) Antagomir-17-5p abolishes the growth of therapy-resistant neuroblastoma through p21 and BIM. PLoS One 3: e2236.

30. Li H, Bian C, Liao L, Li J, Zhao RC miR-17-5p promotes human breast cancer cell migration and invasion through suppression of HBP1. Breast Cancer Res Treat.

31. Matsubara H, Takeuchi T, Nishikawa E, Yanagisawa K, Hayashita Y, et al. (2007) Apoptosis induction by antisense oligonucleotides against miR-17-5p and miR-20a in lung cancers overexpressing miR-17-92. Oncogene 26: 6099-6105.

32. Pickering MT, Stadler BM, Kowalik TF (2009) miR-17 and miR-20a temper an E2F1-induced G1 checkpoint to regulate cell cycle progression. Oncogene 28: 140-145.

33. Sun H, Li QW, Lv XY, Ai JZ, Yang QT, et al. (2010) MicroRNA-17 post-transcriptionally regulates polycystic kidney disease-2 gene and promotes cell proliferation. Mol Biol Rep 37: 2951-2958.

34. Taguchi A, Yanagisawa K, Tanaka M, Cao K, Matsuyama Y, et al. (2008) Identification of hypoxia-inducible factor-1 alpha as a novel target for miR-17-92 microRNA cluster. Cancer Res 68: 5540-5545.

35. Takakura S, Mitsutake N, Nakashima M, Namba H, Saenko VA, et al. (2008) Oncogenic role of miR-17-92 cluster in anaplastic thyroid cancer cells. Cancer Sci 99: 1147-1154.

36. Yang F, Yin Y, Wang F, Wang Y, Zhang L, et al. (2010) miR-17-5p Promotes migration of human hepatocellular carcinoma cells through the p38 mitogen-activated protein kinase-heat shock protein 27 pathway. Hepatology 51: 1614-1623.

37. Liu X, Sempere LF, Ouyang H, Memoli VA, Andrew AS, et al. (2010) MicroRNA-31 functions as an oncogenic microRNA in mouse and human lung cancer cells by repressing specific tumor suppressors. J Clin Invest 120: 1298-1309.

38. Liu CJ, Tsai MM, Hung PS, Kao SY, Liu TY, et al. (2010) miR-31 ablates expression of the HIF regulatory factor FIH to activate the HIF pathway in head and neck carcinoma. Cancer Res 70: 1635-1644.

39. Ivanov SV, Goparaju CM, Lopez P, Zavadil J, Toren-Haritan G, et al. (2010) Pro-tumorigenic effects of miR-31 loss in mesothelioma. J Biol Chem 285: 22809-22817.

40. Creighton CJ, Fountain MD, Yu Z, Nagaraja AK, Zhu H, et al. Molecular profiling uncovers a p53-associated role for microRNA-31 in inhibiting the proliferation of serous ovarian carcinomas and other cancers. Cancer Res 70: 1906-1915.

41. Zhang Y, Guo J, Li D, Xiao B, Miao Y, et al. (2010) Down-regulation of miR-31 expression in gastric cancer tissues and its clinical significance. Med Oncol 27: 685-689.

42. Valastyan S, Reinhardt F, Benaich N, Calogrias D, Szasz AM, et al. (2009) A pleiotropically acting microRNA, miR-31, inhibits breast cancer metastasis. Cell 137: 1032-1046.

43. Valastyan S, Chang A, Benaich N, Reinhardt F, Weinberg RA (2010) Concurrent suppression of integrin alpha5, radixin, and RhoA phenocopies the effects of miR-31 on metastasis. Cancer Res 70: 5147-5154.

44. Wong TS, Ho WK, Chan JY, Ng RW, Wei WI (2009) Mature miR-184 and squamous cell carcinoma of the tongue. ScientificWorldJournal 9: 130-132.

45. Wong TS, Liu XB, Wong BY, Ng RW, Yuen AP, et al. (2008) Mature miR-184 as Potential Oncogenic microRNA of Squamous Cell Carcinoma of Tongue. Clin Cancer Res 14: 2588-2592.

46. Foley NH, Bray IM, Tivnan A, Bryan K, Murphy DM, et al. (2010) MicroRNA-184 inhibits neuroblastoma cell survival through targeting the serine/threonine kinase AKT2. Mol Cancer 9: 83.

47. Imam JS, Buddavarapu K, Lee-Chang JS, Ganapathy S, Camosy C, et al. (2010) MicroRNA-185 suppresses tumor growth and progression by targeting the Six1 oncogene in human cancers. Oncogene 29: 4971-4979.

48. Takahashi Y, Forrest AR, Maeno E, Hashimoto T, Daub CO, et al. (2009) MiR-107 and MiR-185 can induce cell cycle arrest in human non small cell lung cancer cell lines. PLoS One 4: e6677.

49. Lee Y, Yang X, Huang Y, Fan H, Zhang Q, et al. (2010) Network modeling identifies molecular functions targeted by miR-204 to suppress head and neck tumor metastasis. PLoS Comput Biol 6: e1000730.
