# Supplementary material for: Regulation of Cancer Aggressive Features in Melanoma Cells by MicroRNAs
Source: PLoS One. 2011 Apr 25;6(4):e18936. doi: 10.1371/journal.pone.0018936 (PMC3081841; doi:10.1371/journal.pone.0018936)
Supplement: Table S2 — Clinical data of all primary melanoma cultures derived from patients. (DOC) [file pone.0018936.s002.doc]

**Supplementary Table S2**

| Patient | Gender | At diagnosis | | | At time of biopsy (Stage IV) | |
| --- | --- | --- | --- | --- | --- | --- |
|  |  | Age | Breslow (mm) | Site of metastasis | Age | Site of removed metastasis |
| 003 | male | 68 | - | LN* | 68 | LN |
| 005 | female | 78 | 14 | LN | 78 | LN |
| 008 | male | 56 | 3.1 | - | 58 | LN |
| 04 | male | 40 | 2.9 | - | 49 | S.C.** |
| 13 | male | 52 | - | LN, S.C. | 53 | Lung |
| 14 | male | 49 | 4.5 | LN | 53 | LN |
| 15 | male | 51 | - | S.C. | 57 | Lung |
| 18 | male | 29 | 4.4 | - | 31 | LN |
| 19 | male | 62 | - | LN | 66 | LN |
| 41 | male | 40 | 1.5 | - | 57 | Lung |
| 42 | male | 40 | 6.7 | - | 41 | S.C. |
| 67 | male | 49 | - | LN | 50 | Lung |
| 72 | male | 41 | 4 | - | 49 | Spleen |
| 73 | male | 25 | 5 | S.C. | 25 | S.C. |
| 76 | female | 25 | 0.55 | - | 29 | LN |

*Lymph node

**Subcutaneous
