# Supplementary material for: Regulation of Cancer Aggressive Features in Melanoma Cells by MicroRNAs
Source: PLoS One. 2011 Apr 25;6(4):e18936. doi: 10.1371/journal.pone.0018936 (PMC3081841; doi:10.1371/journal.pone.0018936)
Supplement: Table S3 — List of primers used to clone the miRNAs examined in this study. (DOC) [file pone.0018936.s003.doc]

**Supplementary Table S3**

|  | Forward | Reverse |
| --- | --- | --- |
| miRNA-17 | gtacgcggccgcgctgaatttgtatggtttatagttgtta | gtgaattcgcaccttagaacaaaaagcact |
| miRNA-31 | gcatgcggccgcatgagtgtgttttccctccc | ggaattcaaatccacatccaaggaagg |
| miRNA-34a | gcatgcggccgctctttcctccccacatttc | ggaattcggtctgggcatctctcg |
| miRNA-184 | gcatgcggccgcacgtccatttacatcttgtcct | ggaattcaggtctcctcctggaatcc |
| miRNA-185 | gcatgcggccgcaaaggcaaggtcacaggtc | ggaattctaaacagatctccggacagc |
| miRNA-204 | gcatgcggccgctaggacagggtgatggaaag | ggaattcttcatgtcatggttatccca |
